# Cooperation in Networks Where the Learning Environment Differs from the Interaction Environment

**DOI:** 10.1371/journal.pone.0090288

**Published:** 2014-03-14

**Authors:** Jianlei Zhang, Chunyan Zhang, Tianguang Chu, Franz J. Weissing

**Affiliations:** 1 State Key Laboratory for Turbulence and Complex Systems, College of Engineering, Peking University, Beijing, China; 2 Network Analysis and Control Group, Institute for Industrial Engineering, University of Groningen, Groningen, The Netherlands; 3 Theoretical Biology Group, Centre for Ecological and Evolutionary Studies, University of Groningen, Groningen, The Netherlands; Universidad Carlos III de Madrid, Spain

## Abstract

We study the evolution of cooperation in a structured population, combining insights from evolutionary game theory and the study of interaction networks. In earlier studies it has been shown that cooperation is difficult to achieve in homogeneous networks, but that cooperation can get established relatively easily when individuals differ largely concerning the number of their interaction partners, such as in scale-free networks. Most of these studies do, however, assume that individuals change their behaviour in response to information they receive on the payoffs of their interaction partners. In real-world situations, subjects do not only learn from their interaction partners, but also from other individuals (e.g. teachers, parents, or friends). Here we investigate the implications of such incongruences between the ‘interaction network’ and the ‘learning network’ for the evolution of cooperation in two paradigm examples, the Prisoner's Dilemma game (PDG) and the Snowdrift game (SDG). Individual-based simulations and an analysis based on pair approximation both reveal that cooperation will be severely inhibited if the learning network is very different from the interaction network. If the two networks overlap, however, cooperation can get established even in case of considerable incongruence between the networks. The simulations confirm that cooperation gets established much more easily if the interaction network is scale-free rather than random-regular. The structure of the learning network has a similar but much weaker effect. Overall we conclude that the distinction between interaction and learning networks deserves more attention since incongruences between these networks can strongly affect both the course and outcome of the evolution of cooperation.

## Introduction

Cooperation is common in humans, but difficult to explain. The reason is that defectors have an intrinsic advantage over cooperators since they can reap the benefits of cooperation without contributing to the costs of cooperation [1], [2]. There is a huge literature on this topic, both in the biological and human sciences [3]–[5]. Two main mechanisms can help to resolve the paradox of cooperation. The first is based on the idea that cooperation is conditional and only directed to individuals that (for whatever reason) have a high tendency to cooperate as well. The second is based on non-random interactions: if the population is structured in such a way that cooperators tend to interact with cooperators while defectors tend to interact with defectors, defection will also in a short-term perspective not be a successful strategy.

Both mechanisms can be studied well in network models, which are based on the idea that individuals interact in local neighbourhoods [6]–[8]. In this framework, population structure is described by an interaction network, the nodes of which represent the individual agents while the links correspond to the possible interactions. A network model typically assumes that at each point in time all agents are endowed with a certain strategy (i.e. they have a certain tendency to cooperate); that the agents interact with their interaction partners, thereby employing their strategy; that this way they accumulate payoffs; and that subsequently they can change their strategy by comparing their own payoffs with the payoffs of their interaction partners. It has been shown that under these assumptions cooperation can get firmly established, even in situations as the Prisoner's Dilemma game where defection is the dominant strategy in a well-mixed population [9], [10]. However, the emergence and spread of cooperation strongly depends on the learning rules governing the change of individual strategies on the basis of payoff comparisons [11], [12] and on the structure of the interaction network [13], [14]. As a rule of thumb, cooperation can get easily off the ground if the interaction network is heterogeneous (as in scale-free networks; [15]), while it will not easily evolve in homogeneous networks (e.g. random-regular networks [6]).

With a few exceptions [16]–[20], most network models implicitly assume that payoff comparisons with one or more interaction partners is the only factor inducing agents to change their strategy. In other words, individuals can only learn from their interaction partners. In reality, however, individuals can also learn from teachers, parents, or peers with whom they not necessarily interact in a cooperation game. Hence, we have to face the possibility that interaction and learning neighbourhoods only partly overlap. Only few studies consider such an incongruence between the interaction and the learning network. For example, Ohtsuki et al. find that breaking the congruence of the interaction network and the learning network undermines the evolution of cooperation [16], [17]. Based on a second modelling study, Wu et al. conclude that cooperation is generally promoted when the learning neighbourhood is larger than the interaction neighbourhoods [20].

In spite of the mentioned progress that has been accumulated, there are situations that still remain less explored. For instance, to our knowledge, previous investigations paid little attention to the topological differences between the two networks. Accepting this point of view, here we perform a systematic study of how the evolution of cooperation is affected by various degrees of incongruences between the interaction and the learning network. To this end, we consider two standard models for cooperative interactions in 2-person games, the Prisoner's Dilemma game (PDG) and the Snowdrift game (SDG) [14], [21]–[23]. Both games exemplify that mutual cooperation does not necessarily correspond to a Nash equilibrium, even though mutual cooperation corresponds to the population state with the highest average payoff. Yet, both games have a very different strategic structure: the PDG is a game with one dominant strategy (defection), while the SDG is an ‘evasion game’ where defection is the best response to cooperation, while cooperation is the best response to defection. Both games are played by agents whose interaction neighbourhood is characterized by an interaction network. Strategy updating occurs like in in earlier models based on payoff comparisons. However, payoffs are compared with individuals from the learning neighbourhood, and the corresponding learning network is not necessarily identical with the interaction network. We systematically change a parameter 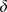, which quantifies the incongruence between the two networks, and ask the question how and to what extent 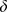 affects the degree of cooperation emerging in the course of time. For both types of networks we consider two variants differing in their degree of heterogeneity: random-regular networks and scale-free networks. As indicated above, cooperation should more easily spread in scale-free networks, but it is not obvious whether the interaction or the learning structure is responsible for that.

## Model Structure

### Overview

To make our results comparable with earlier findings, we largely follow Santos and Pacheco [15] in their assumptions on network construction, accumulation of payoffs and the rules for switching to a new strategy. In our simulations, we consider a population of 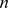 individuals, where 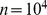 in all results reported. At each point of time, each individual is in one of two states, corresponding to cooperation (*C*) and defection (*D*), respectively. All simulations shown were initialized by assigning a randomly chosen state to each individual, both states having the same probability. In the course of time, these states can change based on payoff-based learning. Time proceeds in discrete steps, each step consisting of an interaction phase followed by a learning phase. Throughout the interaction phase, each individual uses the same strategy (corresponding to its state) in all interactions. This strategy (or state) can only be changed in the learning phase.

The individuals are embedded in an interaction network that characterizes who interacts with whom. In the interaction phase, each individual interacts with all ‘neighbours’ to whom it is linked in the interaction network. Depending on the strategies employed by the interaction partners, each interaction results in a payoff, which can be determined from a payoff matrix (see below). All payoffs thus accrued by an individual 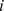 are added, thus yielding a total payoff 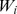 for the interaction phase of the time step.

The interaction phase is followed by a phase of social learning, where individuals can change their state (or strategy) when encountering individuals having achieved a higher payoff during the interaction phase. Individuals encounter such ‘models’ in their learning network. For each individual 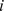, a random model 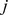 is drawn from those individuals to whom it is linked in the learning network. If the payoff 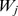 achieved by 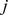 in the interaction phase of the same time step is higher than 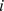
's payoff 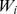, individual i will adopt the strategy of 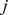 with a probability 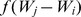 that is an increasing function of the payoff difference 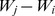 (see [15] for details). Otherwise, focal individual 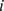 will stick to her previous strategy.

All simulations were run for 11000 time steps. Simulation outcomes such as the average frequency 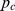 of cooperators were scored by taking the average over the final 1000 steps. Simulations run for much longer time periods revealed that within 10000 time steps stable levels of the relevant variables were reached that remained roughly constant over extensive periods of time. Technically speaking, these stable levels do not correspond to steady states, since in a finite population fixation on either *C* or *D* will eventually occur due to random drift. For practical purposes, this can however be neglected in populations of size 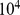 as considered here. Therefore the simulation results obtained after 11000 time steps give a good indication of the balance of selective forces acting on *C* and *D*. All the simulation results reported below are averaged over 100 different realizations of different initial conditions and networks.

### Payoffs

We focus on two paradigm examples for the evolution of cooperation, the Prisoner's Dilemma Game (PDG) and the Snowdrift Game (SDG). In both games, individuals can adopt one of two strategies: cooperation (*C*) or defection (*D*). Cooperation involves some costs, which we normalize to 1. The benefit of cooperation is denoted by 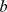. For simplicity, we assume that the payoff in case of mutual defection is 0 for each player. Under these assumptions, 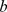 is the only free payoff parameter, and the payoff matrices are given by

**Prisoner's Dilemma Game (PDG):**

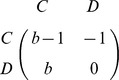



**Snowdrift Game (SDG):**

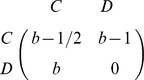



In contrast to the PDG, in the SDG the costs of cooperation are shared by mutually cooperating individuals, and the cooperator receives the benefits of cooperation even in case of being defected. In a one-shot PDG, defection is a dominant strategy and, accordingly, the only Nash equilibrium strategy. In a one-shot SDG with 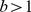, none of the two pure strategies is a Nash equilibrium strategy. Instead, there is mixed Nash equilibrium strategy, which in a well-mixed population corresponds to cooperation with probability 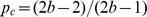.

### Two types of network

Both for interaction and learning networks, we consider two types of network: random-regular networks and scale-free networks. A random regular network [24] is a network whose links are randomly generated but where every node has the same degree 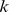 (i.e. the same number of ‘neighbours’). All results reported in this study are based on 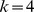. A scale-free network [25] is a network whose degree distribution follows a power law (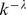), at least asymptotically. Here, for any scale-free network, we first generated a virtual network via the mechanisms of growth and preferential attachment as described in [25] and get its degree sequence. Then, these degrees are randomly given to the nodes of the target network and linked randomly according to the degree sequence. Different from the method in [25], we can generate scale-free networks with the same degree distribution but different links. All results reported in this study are based on 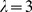, yielding an average degree of 4. We used two different methods to achieve an incongruence 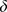 between the interaction and the learning network. These methods will be explained below.

## Simulation Results

### Scenario 1: Overlapping interaction and learning environments

A natural way to study incongruences between interaction and learning neighbourhoods is to assume that individuals base their strategy-updating on payoff comparisons with part of their interaction neighbourhood and some additional individuals outside of this neighbourhood. To model this, we first constructed a random regular interaction network with degree 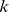. This interaction network served as the starting point for constructing the learning network. For each value of the incongruence parameter 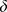 (where 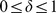) a fraction (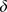) of all connections of the interaction network was randomly discarded. Subsequently, the network was randomly completed again, until a regular network (the learning network) with degree k was obtained. This way, the learning neighbourhood of an individual consists on average of (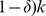 of her interaction partners and 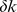 other individuals.

**Figure 1:**
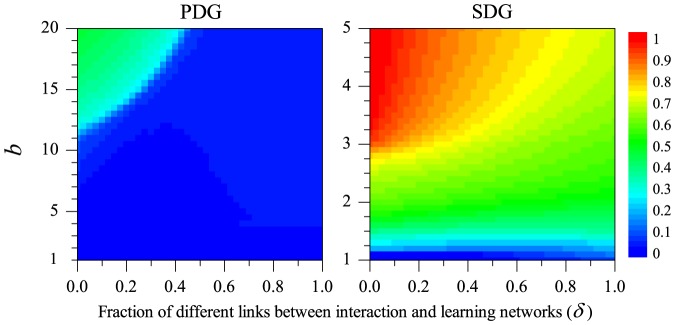
Degree of cooperation achieved in a Prisoner's Dilemma game (PDG, left) and in a Snowdrift game (SDG, right) as a function of the benefit 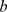 of cooperation and the incongruence 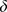 between the interaction and the learning network. The simulation are based on scenario 1, where interaction and learning network overlap and both are random-regular networks with degree 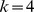.

Fig. 1 illustrates the simulation results. As expected, the frequency of cooperation at steady state is positively related with the benefit 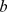 of cooperation. In the PDG (left panel), cooperation only gets a foothold in the population if 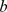 is very high, and even in this case only reaches relatively low frequencies. In the SDG, cooperation reaches appreciable frequencies even at moderate values of 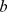, and it often even reaches fixation. The main focus of our study is the effect of the incongruence 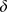 between interaction and learning network on the evolution of cooperation. Fig. 1 clearly shows that the degree of cooperation decreases with 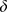. For the range of 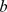-values shown, cooperation in the PDG completely disappeared for 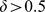, while fixation of cooperation in the SDG did not occur for 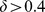. Still, the effect of 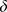 on the evolutionary outcome is not really dramatic: an incongruence of, say, 20% between interaction and learning network (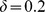) has an effect on the degree of cooperation, but this effect is relatively small when compared to the standard scenario where individuals only learn from their interaction partners (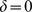).

The above method for constructing two networks with a given degree of incongruence is easily applicable to regular networks, but much less so for other types of network. A certain fraction of connections of the interaction network can of course be discarded for all types of network, but it is not straightforward on how to re-establish links in such a way that a specific type of learning network results. Since we want to study combinations of networks of a given type, we will now address incongruences between interaction and learning network by a different approach.

### Scenario 2: Internal and external learning environments

**Figure 2:**
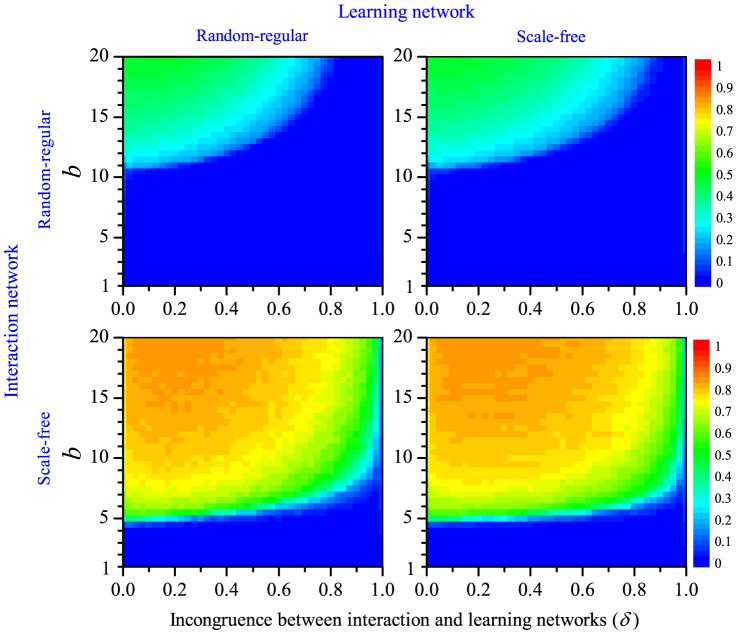
Frequency of cooperation achieved in a Prisoner's Dilemma game as a function of the benefit 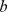 of cooperation and the incongruence 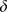 between the interaction and the external learning network. The simulations are based on scenario 2. Both networks can either be random-regular or scale-free. Cooperation is strongly favoured when the interaction network is scale-free (bottom row) and weakly favoured when the external learning network is scale-free (right column).

**Figure 3:**
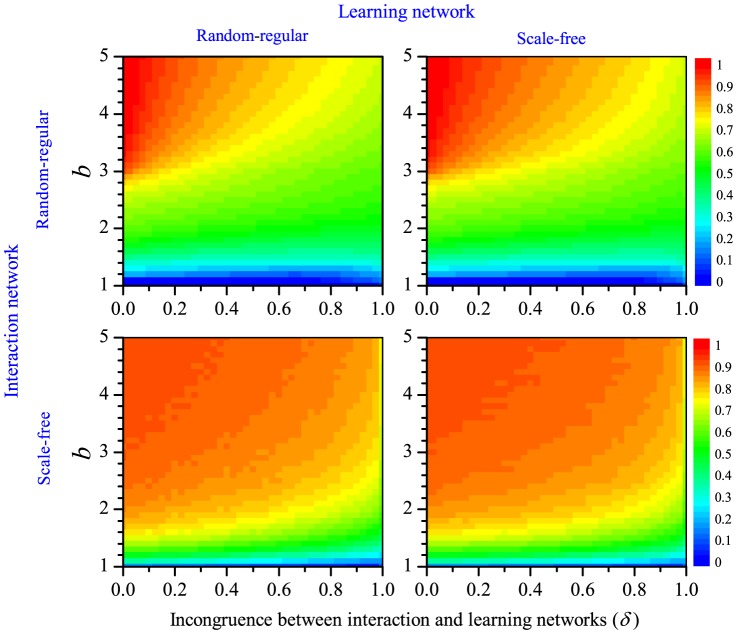
Frequency of cooperation achieved in a Snowdrift game as a function of the benefit 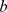 of cooperation and the incongruence 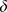 between the interaction and the external learning network. The simulations are based on scenario 2. Both networks can either be random-regular or scale-free. Cooperation is strongly favoured when the interaction network is scale-free (bottom row) and weakly favoured when the external learning network is scale-free (right column).

In a second scenario, we start with two networks that are created separately. The first of these networks is the interaction network, while the second network corresponds to the additional sources of information individuals might use for updating their strategies (e.g. teachers, parents, peers). This second network will be called the ‘external learning network’, while the ‘internal learning network’ is identical with the interaction network. In scenario 2, payoffs are accrued due to interactions in the interaction network. Payoff-based learning takes place as follows: with probability 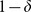 individuals base their choice on whether to switch to another strategy on the payoff comparison with a randomly chosen member of their internal learning neighbourhood (i.e., with a randomly chosen interaction partner); with probability 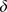 the payoff comparison is being made with a member of the external learning neighbourhood. Since both networks are generated separately, we can now consider various combinations of regular random and scale-free networks. The simulation results for these combinations are illustrated in Fig. 2 for the PDG and in Fig. 3 for the SDG.

Let us first consider Fig. 2. The upper left panel corresponds to a situation where both the interaction network and the external learning network are random-regular networks with degree 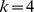. Not surprisingly, the outcome resembles that in the left panel of Fig. 1, where both the interaction and the learning network were also random-regular with the same degree. Yet, cooperation is achieved under a broader range of 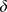-values in scenario 2 than in scenario 1. This can be explained as follows. Take for example the case 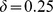, where on average one learning event takes place outside the interaction network. In scenario 1 (Fig. 1), on average three of the 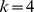 interaction partners are ‘earmarked’ as learning partners, while each individual has on average one additional (fixed) learning partner. In scenario 2 (Fig. 2) all four interaction partners can act as learning partners (in case of internal learning), while there are four different learning partners in case of external learning. We presume that the possibility of payoff-based learning with all interaction partners is mainly responsible for the fact that cooperation is more easily achieved in scenario 2. This does not only apply to the PDG but also to the SDG (compare the left panel of Fig. 1 with the upper right panel of Fig. 3).

In all four panels of Fig. 2 and Fig. 3 the frequency of cooperation is positively related to the benefits 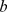 of cooperation and negatively related to the incongruence 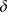 between interaction and learning environments. In addition, the type of network has a marked effect on the evolution of cooperation. In both games, a much higher frequency of cooperation is achieved when the interaction network is scale-free than when it is random-regular. This is fully in line with earlier results indicating that cooperation is favoured by network heterogeneity [6], [13], [14], [26]–[28]. The structure of the external learning network has a similar - be it markedly weaker - effect: for the same values of the parameters 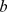 and 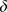 a higher frequency of cooperation is achieved when the external learning network is scale-free than when it is random regular. If both networks are scale-free, cooperators can dominate the population (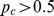) in the PDG even for a high degree of incongruence (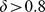), while this never happened even for high values of 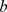 and in the absence of incongruence (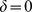) when the networks were random-regular.

Qualitatively, the same conclusions can be drawn as for scenario 1: incongruences between the interaction and the learning network are unfavourable for the establishment of cooperation, but the effect is mainly noticeable in case of strong incongruence. In fact, in case of scale-free interaction networks the incongruence has been quite large (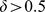) before ‘outside learning’ has a strong effect on the evolution of cooperation.

## Analytical Results: Pair Approximation Dynamics

Since it is useful to complement individual-based simulations with a mathematical analysis, we will now extend the pair approximation method, which has successfully been applied in the special case where the learning network is identical with the interaction network [23], [29]. The pair approximation method tracks the frequency distribution of all possible strategy pairs 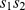 (where 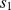 and 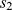 are either cooperation 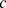 or defection 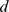), that is the frequency of all network links where one player employs strategy 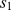 while the other player employs strategy 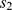. This way, the method accounts for at least some of the spatial structure emerging in a network.

We apply the pair approximation method to the special case where the interaction and the learning network are both random-regular, and where learning individuals learn from a randomly chosen interaction partner with probability 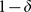 and from a randomly chosen individual from the (external) learning network with probability 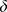. Hence the approach taken corresponds to scenario 2 considered above. Moreover, we derive the equations for the special case 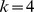, but we include 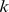 in the equations in order to make them more transparent.

Let 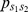 denote the expected frequency of 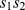 pairs (where 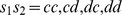) in a population. Accordingly the frequency of cooperators and defectors are given by 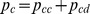 and 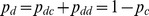, respectively. Following the treatment of Hauert and Doebeli (see the supplementary information to [23]), we will now derive differential equations for the change in 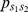 over time. A change in strategy pairs can only occur in the event that a player (let us call her 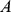) changes her strategy as the result of learning from another player 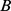. Such a change in strategy can only occur if the two players differ in strategy, that is, if either 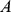 used *C* and 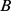 used *D* or vice versa. The probability that a potential learning event takes place in such a configuration is in both cases given by 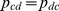. The rate of change of 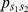 due to such learning event is given by this probability times the probability that player 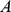 adopts player 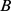
's behaviour times the net change in the number of 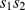 pairs caused by the switch in 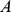
's behaviour. As indicated in the Overview section above, the probability that 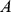 adopts 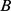
's behaviour is given by 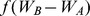, where 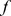 is an increasing function of the payoff difference between players 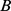 and 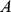. We will now consider four different cases.

**Figure 4:**
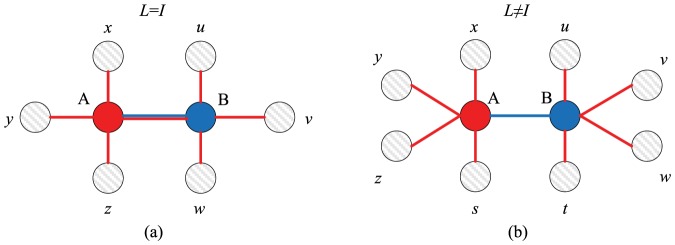
Diagrams illustrating a potential learning event. In (a) the focal individual 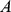 learns from an individual 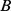 that is part of 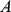
's interaction network (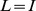). Since 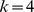, both 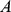 and 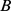 have three other interaction partners, whose strategy (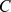 or 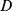) is indicated by 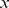, 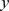, 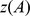 and 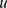, 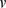, 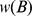, respectively. In (b) 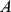 learns from an individual 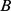 that does not belong to 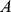
's interaction network (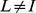). Now both 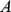 and 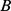 have four different interaction partners.

(a) 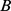 belongs to the interaction network of 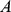 (which we symbolize by 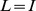); 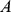 used *C* and 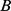 used *D* in the interaction phase. As indicated in Fig. 4(a) defector 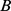 had one cooperating neighbour (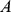) and three other neighbours with strategies 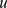, 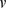 and 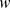. Each of these strategies is either *C* (with conditional probability 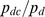) or *D* (with conditional probability 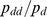). The payoff of 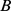 is given by 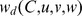, which indicates the payoff of a defector confronted with the given configuration of neighbours. Similarly, 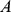 had one defecting neighbour (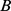) and three other neighbours employing strategies 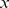, 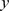, and 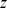. These strategies are either *C* (with conditional probability 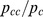) or *D* (with conditional probability 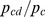), and the payoff of cooperator 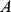 is given by 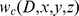. For each neighbour configuration, player 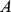 will switch from *C* to *D* with probability 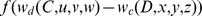. Let 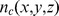 denotes the number of cooperators among those neighbours of 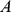 that are not identical with 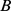. Then 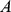 was involved in 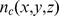 *CC*-pairs and in 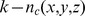 *CD*-pairs before the change in behaviour. By switching from *C* to *D*, this changes into 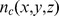 *CD*-pairs and in 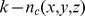 *DD*-pairs. Hence the change in 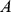
's behaviour results in a loss of 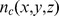 *CC*-pairs and a net change of 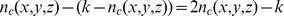 *CD*-pairs. Since we distinguish between 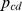 and 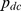 (in line with [23]), half of the change in *CD*-pairs (i.e. 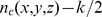) ascribed to the configuration *CD* and half to the configuration *DC*. Summarizing all this, the expected change in the frequencies of *CC* and *CD* pairs due to a potential learning event of a cooperator 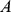 confronted with a defector 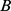 is given by:

Change in 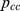:
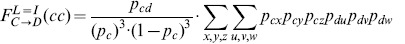


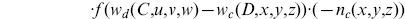



Change in 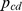:
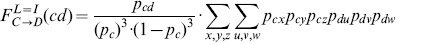


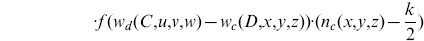



(b) 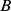 does again belong to the interaction network of 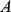 (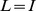), but now 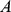 used *D* and 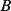 used *C* in the interaction phase. The calculations are completely analogous to case (a) above. Now defector 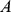 had 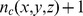 cooperating neighbours and was therefore involved in 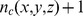 *DC*-pairs and in 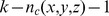 *DD*-pairs during the interaction phase. By switching from *D* to *C*, this changes into 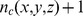 *CC*-pairs and in 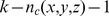 *CD*-pairs. Hence the change in 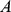
's behaviour results in a gain of 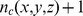 *CC*-pairs and a net change of 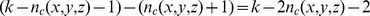 *CD*-pairs. As before, half of the latter change (i.e. 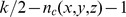) is ascribed to the configuration *CD* and half to the configuration *DC*. Taken together, all this results in:

Change in 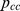:
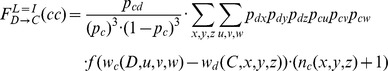



Change in 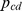:
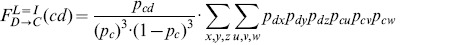


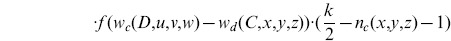



(c) Now 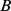 does no longer belong to the interaction network of 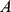 (which we symbolize by 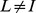); 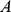 used *C* and 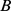 used *D* in the interaction phase. The configuration 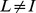 is illustrated in Fig. 4(b): 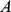 and 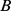 are no longer interaction partners and instead have interaction partners playing strategies 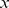, 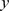, 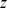, 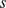 (player 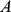) and 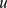, 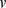, 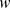, 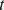 (player 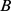), respectively. Consider again the case that 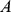 used *C* and 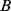 used *D* in the interaction phase. When 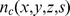 denotes the number of cooperating interaction partners of 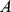, 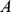 was represented in 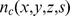 *CC*-pairs and in 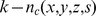 *CD*-pairs. If 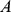 switches from *C* to *D*, this results in 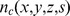 *CD*-pairs and in 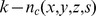 *DD*-pairs. Hence the change in 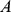
's behaviour results in a loss of 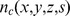 *CC*-pairs and a net change of 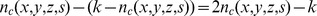 *CD*-pairs. As above, we can now summarize the expected change in the frequencies of *CC* and *CD* pairs due to a potential learning event of a cooperator 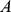 confronted with a defector 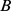 who not interacted with 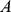:

Change in 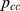:
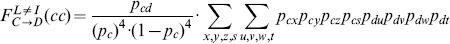


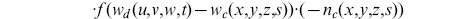



Change in 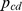:
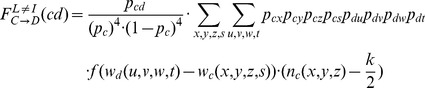



(d) 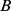 does not belong to the interaction network of 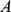 (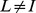), but now 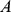 used *D* and 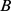 used *C* in the interaction phase. Completely analogous calculations to those before yield:

Change in 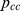:
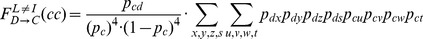


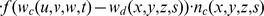



Change in 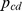:
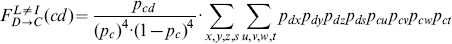


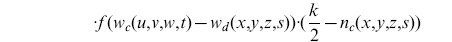



Taking all four cases together and considering that 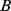 belongs to 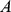
's interaction network (cases (a) and (b)) with probability 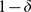, while 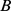 is external to 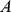
's interaction network (cases (c) and (d)) with probability 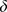, we now have derived the desired system of differential equations:
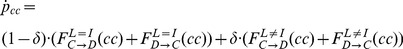
(1)

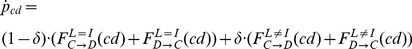
(2)


Taking into consideration the symmetry condition 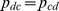, plus the constraint the constraint 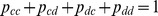, the above equations can be treated by setting 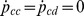 and solving for 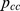 and 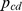, thus the equilibrium density of cooperators can be obtained from 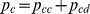.

**Figure 5:**
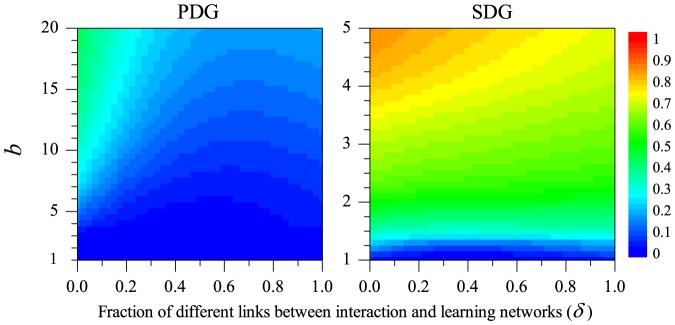
Equilibrium level of cooperation as predicted by the analytical pair approximation method. As before, the degree of cooperation achieved in a Prisoner's Dilemma game (PDG, left) and in a Snowdrift game (SDG, right) is shown as a function of the benefit 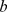 of cooperation and the incongruence 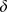 between the interaction and the learning network. Since the pair approximation method is based on scenario 2, the panels should be compared with the simulation results shown in the upper left panels of Figs 2 and 3, respectively.

Thus, we can investigate how cooperation is affected by the incongruence between networks, 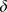, and by the main payoff parameter 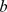. As illustrated by Fig. 5, the pair approximation approach yields qualitatively the same conclusions as our earlier simulations: cooperation is favoured by large values of 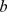 but hampered by a larger incongruence between the learning and the interaction network. Quantitatively, the pair approximation method predicts a lower degree of cooperation than the simulations. This is understandable, since the evolution and maintenance of cooperation reflects the emergence of spatial structure (clusters of cooperators). This structure can be potentially quite rich, and only part of it may be captured by the pair approximation method.

## General Conclusions

In this paper, we aimed to investigate the influence of incongruence between the interaction network and learning network on the cooperation evolution. In both the PDG and the SDG it turned out that cooperation is hampered if these two networks do not coincide. This is easy to understand: cooperation can be maintained once clusters of cooperative individuals have formed. Individuals from such a cluster will only change her strategy if they encounter a defector, and such a change is unlikely unless the defector has a high payoff. If the individuals of a cluster of cooperators learn from each other, they are not inclined to change their strategy, since they will not meet defectors. This is different if these individuals can also learn from ‘outsiders’. Once one individual in a cluster of cooperators has switched to defection, this can have a snowball effect, since this individual can serve as a model for its neighbours as well. In view of this, the most interesting conclusion of our study is perhaps that a rather strong incongruence between the networks is required before the degree of cooperation drops to considerably lower levels.

For the standard model where individuals learn from their interaction partners it is well established that the type of network has a considerable effect on the degree of cooperation. In general, cooperation can be more easily achieved in heterogeneous networks (like scale-free networks) than in homogenous networks (like random regular networks) [15]. Our results confirm this finding and indicate that the heterogeneity of the interaction network is much more important than the heterogeneity of the learning network. In both kinds of network, a switch from a random regular network to a scale-free network results in a higher degree of cooperation, but the effect size is much larger when the interaction network is more heterogeneous than when the learning network is more heterogeneous.
